# Patient and clinician opinions of patient reported outcome measures (PROMs) in the management of patients with rare diseases: a qualitative study

**DOI:** 10.1186/s12955-020-01438-5

**Published:** 2020-06-10

**Authors:** Olalekan Lee Aiyegbusi, Fatima Isa, Derek Kyte, Tanya Pankhurst, Larissa Kerecuk, James Ferguson, Graham Lipkin, Melanie Calvert

**Affiliations:** 1 Centre for Patient-Reported Outcomes Research, College of Medical and Dental Sciences, University of Birmingham, Birmingham, UK; 2 Institute of Applied Health Research, University of Birmingham, Birmingham, UK; 3 NIHR Birmingham Biomedical Research Centre, University Hospitals Birmingham NHS Foundation Trust and University of Birmingham, Birmingham, UK; 4 NHS England and NHS Improvement, Birmingham, UK; 5 University Hospitals Birmingham NHS Foundation Trust, Queen Elizabeth Hospital Birmingham, Birmingham, UK; 6 Birmingham Women’s and Children’s NHS Foundation Trust, Birmingham, UK

**Keywords:** Rare diseases, Rare disorders, Patient involvement, Qualitative study, Health-related quality of life, HRQOL, Primary sclerosing cholangitis, Cholestasis, Patient reported outcome measures, PROMs, ePROMs, Renal transplant, Transitioning

## Abstract

**Background:**

Rare diseases may be life-threatening or chronically debilitating conditions. Patient care needs are often complex and challenging to coordinate and deliver effectively. Rare diseases and their clinical management may therefore substantially impact on patients’ health-related quality of life (HRQOL). The use of patient-reported outcome measures (PROMs) may complement clinical assessments by elucidating patients’ perspectives on their health status and care priorities. This study explored the opinions of patients and clinicians on the use of PROMs in the management of patients with rare diseases in routine clinical practice.

**Methods:**

A total of 15 semi-structured one-to-one interviews were conducted with four patients with primary sclerosing cholangitis (PSC); five renal transplant recipients; and six PSC doctors from University Hospitals Birmingham (UHB) NHS Foundation Trust. A focus group session was also conducted with 10 clinical staff members (doctors, nurses and other allied health professionals from UHB). The suitability and acceptability of the Chronic Liver Disease Questionnaire (CLDQ) and the Short Form 12 (SF12) were assessed by patients with PSC and their doctors while the Paediatric quality of life inventory Transplant Module (PedsQL-TM) and the EuroQoL-5 dimensions (EQ. 5D) were evaluated by the renal transplant recipients and their doctors. The discussions were audio recorded and transcribed verbatim. Coding of the transcripts was done using the Nvivo 11 Plus software. Thematic analysis was conducted to identify the main themes and subthemes.

**Results:**

Four themes were identified, namely: (i) potential benefits of PROMs in the management of rare diseases; (ii) views on selected questionnaires; (iii) practical considerations for implementation; and (iv) potential facilitators and barriers of implementation. Patients and clinicians suggested that the use of ePROMs may facilitate patient-centred care by promoting patient-clinician communication, highlighting aspects of HRQOL that are important to patients and encouraging patient involvement in their care. They also felt that the disease-specific CLDQ and PedsQL-TM were more relevant than the generic SF12 and EQ-5D.

**Conclusions:**

Patients with rare diseases often experience impaired HRQOL. The use of an ePROM system may enhance the routine management of patients with rare diseases.

## Background

According to the European Union criterion, a ‘rare’ disease is one that has a prevalence of 5 or less cases per 10,000 of the general population; while in the United States, a disease is considered ‘rare’ if it affects less than 200,000 people [1, 2]. In the UK, it is estimated that over three million people will be affected by a rare disease at some point in their lives [2]. Due to the small numbers of patients living with each type of rare disease, there is often limited awareness, knowledge and experience of managing those with the condition [2, 3].

Given these challenges, capturing the perspectives of patients with rare diseases is particularly important as it may provide a better picture of patients’ health status and the outcomes of care they value. This information could assist with the prioritization of care outcomes [4]. Patient perspectives may be captured using patient-reported outcomes (PROs): defined as “any report of the status of a patient’s health condition that comes directly from the patient, without interpretation of the patient’s response by a clinician or anyone else.” [5] PRO data is captured using questionnaires known as patient-reported outcome measures (PROMs) [5]. By providing patients’ perspectives of their health status, the use of PROMs may facilitate a better understanding of the physical and psychosocial impact of disease and treatment on patients’ health-related quality of life (HRQOL) and symptoms [4, 6]. Ideally, PROMs should be developed with patients and clinicians using a combination of qualitative and quantitative methods. An initial long form which addresses all relevant domains is usually generated through literature reviews and discussions with the patients and clinicians [7]. This initial version is then revised and validated. During the validation process, poorly performing questions are identified and removed resulting in a shorter and more valid PROM [8].

PROMs need to be validated to ensure that they actually measure what they are supposed to measure, produce consistent results and capture information that matter to the target population [9].

PROMs may be collected electronically as ePROMs, which allow real-time communication of data. The routine collection of ePROMs can assist clinicians with: the monitoring of patient symptoms; the identification of unmet needs and concerns; the prioritization and/or the tailoring of treatment to the needs of individual patients; and ultimately lead to improvements in the quality of patient care [6].

The aim of this qualitative study was to explore patient and clinician views on the potential role for PROMs/ePROMs in the routine management of rare disease based on renal transplant recipients transitioning from paediatric to adult services and patients with primary sclerosing cholangitis (PSC). Summaries of the pathology and epidemiology of these disease groups are provided in Tables 1 and 2 respectively.

**Table 1:** Renal transplantation

A number of rare conditions such as renal dysplasia, cystinosis, focal segmental glomerulosclerosis, reflux nephropathy, post urethral valves and nephronophthisis may lead to babies being born with poorly developed kidneys or developing significant kidney damage in childhood [10]. These patients may require a renal transplant at some point to improve their chances of survival and quality of life [11].	
In the UK, the five-year graft survival rates following the first adult deceased and live donor kidney transplant are 86 and 92% respectively [12].	
However, coping with the demands of preserving a renal transplant can be challenging for adolescents especially during the transition from paediatric to adult care [13]. There is a high rate of graft failure and acute or chronic rejection due to poor adherence to prescribed medical regimen [14]. Studies have found that the transitioning process may increase the risk of allograft loss [15]. Rejection episodes may be life threatening to patients and a significant burden to the health system [16].

**Table 2:** Primary sclerosing cholangitis

Primary sclerosing cholangitis (PSC) is a progressive disease of the liver and gallbladder characterized by inflammation and scarring of the bile ducts [17]. This may lead to the accumulation of bile which in turn may lead to liver damage and in the long term, cirrhosis, portal hypertension, liver tumours and liver failure [17].	
While PSC may occur at any age, it is more frequent in adults 30–60 years old. PSC is approximately twice as common in men [18]. It is estimated that 70–80% of patients with PSC have inflammatory bowel diseases such as ulcerative colitis and Crohn’s disease [19].	
PSC is associated with a high level of morbidity which may have a significant impact on HRQOL [20]. Tiredness or fatigue may be an early symptom while pruritus, jaundice, abdominal pain, weight loss, fevers, hyperpigmentation, vitamin deficiencies and metabolic bone disease may occur as disease progresses [21].	
The aim of medical treatment is to control symptoms and manage any complications. Currently, the only clinically proven treatment for severely damaged liver is transplantation [17]. However, after transplantation, there is a likelihood of recurrence and acute rejection [22].	

## Methods

This study was reported following the consolidated criteria for reporting qualitative research (COREQ) guideline [23].

### Setting

The setting for this study was the Centre for Rare Disease Studies (CRDS), Birmingham [24]. The centre was set up to: (i) serve as a supra-regional hub bringing together multi-speciality and multi-disciplinary teams to deliver highly-organised care to patients with rare diseases [24]. (ii) support basic and applied research that would improve the diagnosis and clinical management of these disorders [24].

### Participants

Renal transplant recipients aged 16–25 years old (the age range for the transition from paediatric to adult care) and patients with PSC aged 18–80 years old were recruited using a purposive sampling approach. Eligible patients from both patient groups were approached in clinic by medical personnel and provided participant information sheets for the study (see Supplementary material S3). Whilst the CRDS manages a variety of patients with rare diseases, the decision was made to focus on these two patient groups as they provided a larger pool of patients to recruit from. All participants were required to have the capacity to read and write, in order to be eligible for this study. Doctors and nurses at the CRDS involved in the care of renal transplant recipients and or PSC patients were eligible for the study. Written informed consent was collected from all participants.

### Data collection

The only focus group discussion held as part of this study was conducted with the renal multi-disciplinary team (MDT). This was moderated by non-clinical research fellows, Drs Fatima Isa (FI) and Tom Keeley (TK). The aim was to promote group discussion and allow an assessment of the degree of consensus on the topics of discussion [25]. FI conducted one-to-one interviews with PSC clinicians as a mutually convenient date and time was not found for a focus group discussion for them. FI only conducted one-to-one interviews with the patients to encourage open discussions of potentially sensitive illness experiences [26].

Participants were asked for their opinions on four validated PROMs commonly used in research involving these patient groups. The Chronic Liver Disease Questionnaire [27] (CLDQ) and the Short Form 12 [28] (SF12) were given to patients with PSC and their doctors while the Paediatric quality of life inventory Transplant Module (PedsQL-TM) [29] and the EuroQoL-5 dimensions (EQ. 5D) [30] were evaluated by transplant recipients and their doctors. Table 3 presents a brief description of these PROMs.

**Table 3:** Description of measures evaluated by study participants

Measure	Description
Medical Outcomes Study Short-Form 12 (SF-12)	A 12-item generic HRQOL measure derived from the SF-36 [31, 32]. There are 8 dimensions namely: (i) physical functioning (ii) physical role (iii) bodily pain (iv) general health (v) vitality (vi) social functioning (vii) emotional role (viii) mental health. These can be computed into 2 distinct clusters, PCS-12 and MCS-12 with higher values indicating better HRQOL [31].
EuroQOL 5-dimension (EQ-5D)	A utility measure with a self-classifier and a visual analogue scale (VAS) which can be used to value health states [33]. The self-classifier includes 5 dimensions: (i) mobility (ii) self-care (iii) usual activities (iv) pain/discomfort (v) anxiety/depression. Each dimension has 3 levels of severity (no problems, some problems, and severe problems) and it is possible to describe 243 health states between 0 (dead) and 1 (perfect health) [33].
Chronic Liver Disease Questionnaire (CLDQ)	A 29-item liver disease–specific HRQOL questionnaire with 6 dimensions [27] namely: (i) abdominal symptoms (ii) activity (iii) emotional function (iv) fatigue (v) systemic symptoms (vi) worry. Summary scores for each domain range from 1 (most impairment) to 7 (least impairment).
Paediatric Quality of Life Inventory - Transplant Module version 3.0 (PedsQL-TM 3.0)	A 46-item transplant specific module of the PedsQL questionnaire [34] with 8 dimensions namely: 1) about my medicines I (barriers to medical regimen adherence), 2) about my medicines II (medication side effects), 3) my transplant and others (social relationships and transplant), 4) pain and hurt (physical discomfort), 5) worry (worries related to health status), 6) treatment anxiety (fears regarding medical procedures), 7) how I look (impact of transplant on appearance), and 8) communication (communication with medical personnel and others regarding transplant issues) [34]. A 5-point response scale is utilized Items are reverse-scored and linearly transformed to a 0 to 100 scale (0 = 100, 1 = 75, 2 = 50, 3 = 25, 4 = 0) with higher scores indicating better HRQOL [35].

Interview guides were used for the focus group and interviews (see Supplementary material S1 and S2). These were informed by existing literature and discussions with the research team [36]. The focus group discussion and interviews were audio recorded and lasted for 30–60 min. The digital files were transcribed verbatim and personal details were anonymised to preserve the identity of participants.

### Data analysis

Inductive thematic analysis was conducted as proposed by Braun and Clarke [37]. Early transcripts were read repeatedly by FI and OLA to aid familiarisation and assist with development of codes [38]. The transcripts were imported to QSR Nvivo 11 for data management and preliminary codes were developed. These were grouped into potentially relevant themes based on logic and reviewed with members of the research team (MC/DK). Further analysis clarified the specific nature, categorisation and description of each theme. Extracts were selected from the transcripts to illustrate the themes and sub-themes. Data saturation was not a goal for this study, as we did not anticipate recruiting sufficient patient participants due to the rare nature of the conditions [39]. Rather, we sought to explore patient and clinician views on the potential role for PROMs/ePROMs in the routine management of rare diseases.

## Results

A total of 15 one-to-one interviews were conducted. There were four with patients with PSC; five with renal transplant recipients; and six with PSC clinicians (three consultant hepatologists, a registrar, a clinical lecturer and a specialist nurse). The focus group discussion with the renal multidisciplinary team (MDT) included 10 participants comprising a: youth worker, renal registrar, support worker, staff member from Birmingham Children’s Transplant and Transition, renal transplant coordinator, psychologist, renal rare disease specialist nurse, advanced nurse practitioner in renal transplantation and two kidney specialists.

**Fig. 1:**
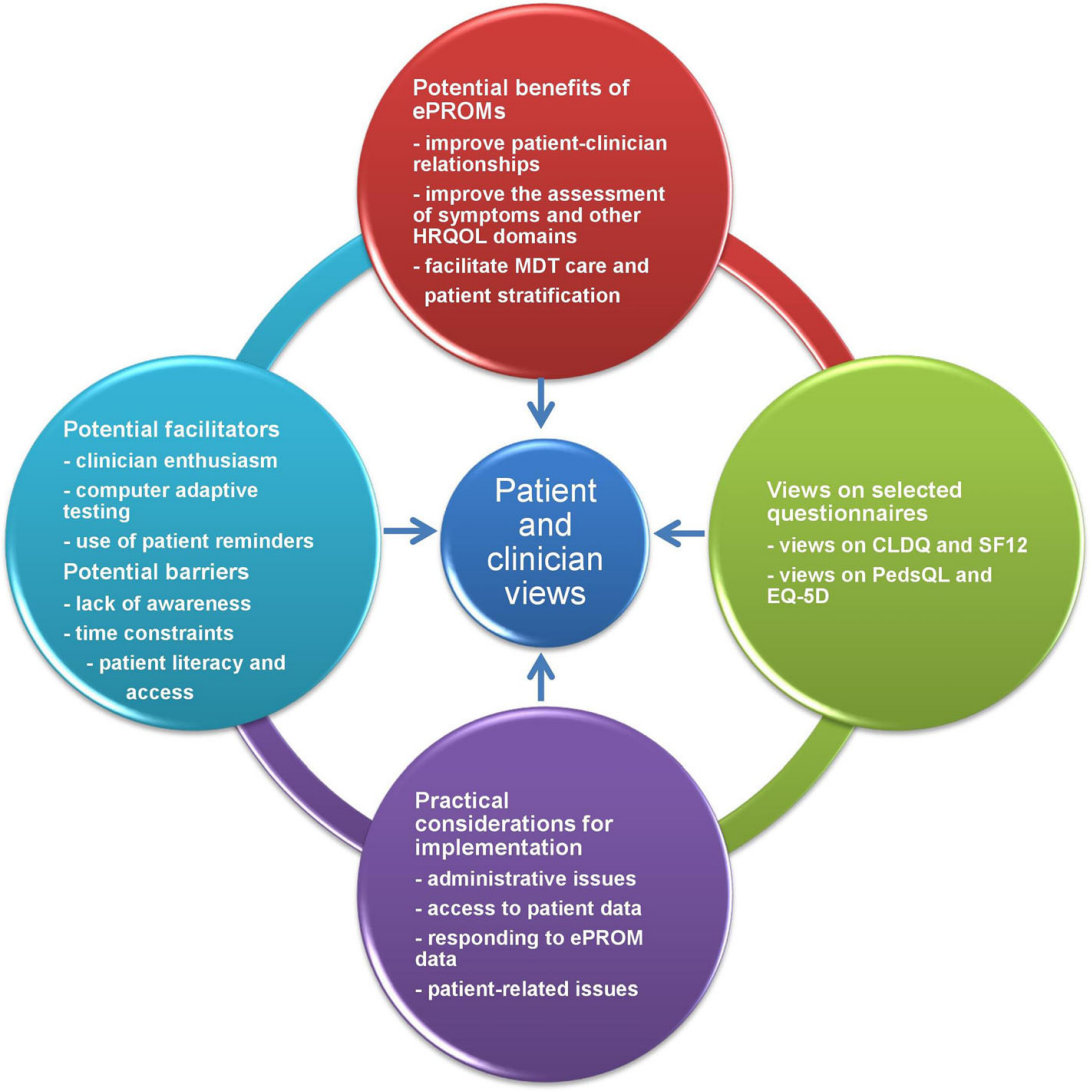
Thematic schema

Four themes were identified, namely: (i) potential benefits of PROMs in the management of rare diseases; (ii) views on selected questionnaires; (iii) practical considerations for implementation; and (iv) potential facilitators and barriers of implementation. Fig. 1 illustrates the relationships between the themes and patient and clinician views. These are discussed in detail below with illustrative participant quotes. Further details are provided in supplementary Tables S1, S2, S3 and S4.

### Potential benefits of PROMs in the management of rare diseases

Within this theme, participants outlined their views regarding the potential benefits of using PROMs in the routine clinical management of patients with rare disease. Patients and clinicians first discussed three potential settings in which PROMs may be used, namely: face-to-face clinical consultations, patient reviews between hospital appointments and virtual clinic consultations. While all the patients were in favour of using PROMs in the first two settings, there were mixed views about the idea of virtual clinics with or without PROMs. These patients strongly preferred face-to-face consultations believing that they promoted a sense of connection with their doctor.

*“Yeah, that’s what I’m kind of suggesting as well so like I said if you fill it in before then you can do it, discuss it in the consultation … .” (PSC patient 4)*

*“….you don’t even have to assess that information in clinic because that will take some time but between clinics or post clinic at some point. I am sure clinicians do that.” (PSC patient 1)*

*“No I feel like that would be stretching it too much, I feel like the Doctors do need to see you regularly. I feel like you need to have that connection with your Consultant because he’s there for you” (Renal transplant patient 1)*

### Potential benefits of PROMs

One patient suggested that PROMs could provide clinicians an insight into their lived experiences of disease.

*“So I feel like it’s really good and like I feel like, if you get more people to fill it in, you’d know how it is for a renal patient live their life … ” (Renal transplant patient 1)*

Clinicians suggested that PROMs could help improve patient-clinician relationships by acting as a conversation starter during clinical consultations, enhancing communication and demonstrating clinician interest and concern to patients.

*“ … It’s always about having a conversation … So I notice here you’ve put ‘I have no problems walking about’. ‘Can you just tell me a little bit more about that?’ … .if we don’t start the conversation from it, we miss the richness that’s going to come from the patient.” (MDT focus group participant)*

*“PROMs are important because it impacts the patient’s journey and a patient should feel that us as clinicians are listening to them and we are listening to what concerns them, not just what we see on a screen and concerns us.” (PSC doctor 1)*

The potential influence of PROMs on patient-clinician interaction was particularly important to clinicians who managed patients transitioning from paediatric to adult care services. Getting this group of patients engaged in their care was reported as challenging and PROMs was considered a tool that may assist with engagement.

*“ … ..so you get in a habit of always discussing their care with the parent because they know exactly how to pinpoint the problem … .where we have to discuss it with a young person, they will give you monotone answers, not even care about discussing their problem … ..” (MDT focus group participant)*

*“(referring to patients) … ..they’ll put things down on a sheet that they won’t actually say to you in person.” (MDT focus group participant)*

A clinician believed the use of PROMs could influence the approach to patient care by enabling clinicians explore other domains of HRQOL such as psychological wellbeing as well as the physical symptoms.

*“Er, it’s asking about mood which I think is something that maybe as clinicians we’re not very good at asking our patients about directly, er, we generally tend to say something you know, how are you feeling and it doesn’t really, we maybe don’t go necessarily down into mood and anxiety so this is a good way of picking.” (PSC doctor 6)*

Clinicians also suggested that PROMs could facilitate multidisciplinary care.

*“It might be a way of getting other allied health professionals on board” (PSC doctor 4)*

A patient suggested that completing a PROM could help patients recall issues they might want to discuss during their clinical consultation.

*“This questionnaire is good. It kind of jogs your memory.” (PSC patient 1)*

Patients and clinicians believed that PROMs could improve symptom reporting and assist with monitoring the evolution of symptoms over time. They also felt PROMs could be used to assess patient response to treatment and the side effects of medication.

*“I think also it would be useful as a tool sort of over time to see how things change for individual patients and to get a better idea of how symptoms progress with time” (PSC doctor 2)*

*“ … ..a lot of the young adults and older adults do have significant side effects from the drugs, even though our objective markers are good. So I think exploring that further, you know could be really very helpful.” (MDT focus group participant)*

A clinician suggested that PROMs may facilitate the stratification of patients so that the most symptomatic patients are seen by consultants while more stable patients are either seen at nurse-led clinics and/or seen less frequently in clinic.

*“You can use it to improve the care of the patients in clinic … you can stratify if patients have a lot of symptom burden or anxiety or something, you can make sure that they’re seen by the consultant. If someone’s very minimally symptomatic and is actually very well, maybe they could have nurse-led care or you know come less frequently” (PSC doctor 3)*

### Views on selected PROMs

#### Views on CLDQ and SF12 (patients with PSC and their clinicians)

Patients and clinicians compared the CLDQ and the SF12. Both were of the opinion that the CLDQ was acceptable and relevant, covering most PSC symptoms. However some patients pointed out that a patient may experience symptoms included in the questionnaire due to other illnesses and not necessarily PSC. Patients and clinicians considered the SF12 too generic and of limited relevance for PSC patients. A doctor suggested that it could be useful for comparing patients with different medical conditions.

*“(referring to CLDQ) Yeah, I think that one’s quite good because all those questions are quite relevant to the stuff that I kind of sometimes feel.” (PSC patient 4)*

*“…the questionnaire (CLDQ) is fine; it’s just, er, whether, there’s no way that says that we know that it relates to PSC.” (PSC patient 2)*

*“I mean a lot of these things in SF12, apart from question 2 and perhaps 1, a lot of them are related to patient ……..motivation. They don’t really talk about things like cholangitis flares, itching.” (PSC doctor 1)*

*“But it’s very generic (referring to SF12), but it’s then, so it’s very easy for someone to fill this in and for you to then compare this with diabetes, obesity, whatever or healthy people, but it’s not liver and it’s not PSC IBD” (PSC doctor 3)*

Patients and clinicians suggested including a free text box where patients can mention other issues not covered in the questionnaire. PSC doctors suggested that patients would prefer questionnaires tailored to their needs and commented on the absence of direct questions about patient fears.

*“….it is a good start because it gives you a baseline, but if you really wanted to go the whole way, I think patients would like to have something that is specific to them.” (PSC doctor 3)*

*“I think what’s missing is the direct, what is the fear that you have of dying, what’s the fear of you of getting colon cancer, what’s the fear of you getting bile duct cancer, and how does that fear manifest itself?” (PSC doctor 3)*

#### Views on PedsQL and EQ-5D (renal transplant recipients and MDT participants)

Patients and MDT participants thought that the PedsQL would be helpful and relevant as it covered issues frequently discussed in clinic. However, some patients felt the PedsQL was more relevant to recent renal transplant recipients.

*“I feel like this one’s really good because I can relate to so many things in here…” (Renal transplant patient 1)*

*“I think it would be more relevant people who have just recently had their transplants so people who are being discharged from hospital should be given these questionnaires.” (Renal transplant patient 3)*

Patients suggested adding questions that inquire about patient experience of the receiving a transplant. They also suggested including questions that explore family views or a separate questionnaire for family members.

*“See there could be another survey for parents ….basic questions to see how parents feel as well as the patient, I know you’re trying to see how the patient feels but then again the services could also be developed for parents.” (Renal transplant patient 3)*

An MDT participant suggested adding questions that went beyond physical concerns and delved into patient fears about their future. This suggestion echoed the point made by a PSC doctor about the need for questions addressing patient fears of developing other conditions in future.

*“I suppose sort of identity, which is difficult to grasp, and also fears for the future, Will I need a transplant in the future? Will my children get this in the future? Will anybody find me attractive in the future? I know you’ve got visible difference here…I suppose are we meeting their unmet needs?” (MDT focus group participant)*

Concerning the EQ-5D, an MDT participant felt anxiety and depression, which were combined in question number five, were very different concepts. Therefore, responding to the question would be challenging.

*“Certainly with the anxiety and the depression one on the EQ5D, anxiety and depression are two really different things, so if you’re ticking one, is it because you’re anxious, because you’re depressed or actually you don’t really know what you’re feeling?” (MDT focus group participant)*

### Practical considerations for implementation

Patients and clinicians discussed numerous practical issues they believed required consideration/addressing for PROMs to be implemented successfully in clinical practice.

(i) Administrative issues

One of the issues considered was the mode of administration. Patients and clinicians compared electronic and paper formats of PROMs, weighed the pros and cons of each and then expressed their preferences. Some participants believed completing electronic questionnaires using mobile devices and tablets was quick, easier to manage and environmentally friendly while others felt that paper questionnaires would give them the chance to provide more information. All the clinicians preferred an electronic format while three out of eight patients preferred paper questionnaires. One patient was indifferent while another expressed a preference for face-to-face conversation despite choosing electronic questionnaires.

*“I don’t mind paper, but I would prefer electronically. First of all, it’s environmentally-friendly and – it’s also much better to keep the record electronically, isn’t it? less hassle, it’s much quicker, it’s more efficient, more accurate…” (PSC patient 3)*

*“I think paper based, yeah…” (Transplant patient 1)*

*“I think they’re a lot more engaging on an iPad, yeah. I just think they’re a bit more … I honestly think they hate pen and paper” (MDT focus group participant)*

A majority of the study participants felt that the local myHealth@QEHB patient portal was suitable and acceptable platform for delivery of ePROMs. myHealth@QEHB is a secure electronic patient records portal developed by the in-house Technical Development and Informatics team at University Hospitals Birmingham NHS Foundation Trust (UHB) [40]. It allows patients in long-term care to remotely access much of their clinical information held at the hospital, including their letters and laboratory results [40].

However one clinician complained that the 2-part patient registration process was cumbersome.

*“So we could do it through MyHealth [patient portal], it’s just a bit of a faff for us to do this in the clinic because the patient has to have set up their MyHealth account and then remember their log-in.” (MDT focus group participant)*

Concerning the frequency of administration, participants suggested time intervals ranging from every three months to yearly. The general opinion was that the timing should coincide with the existing frequency of follow-ups appointments.

*“… Yeah, I come in every year anyway so I’d be good to do it every year and then you can see what happened last year to the next year can’t you? If it’s getting worse or better…” (PSC patient 4)*

Participants compared the option of patients completing their PROMs in the waiting room just before their clinic appointments to completion at home prior to their appointment dates. Participants acknowledged that completion in the waiting room had the benefit of providing patients with something to do whilst waiting to be seen. Participants thought that completion of questionnaires at home, well ahead of clinic appointments, meant that patients could do so in the comfort of their own homes, without the stress associated with being in clinic. It also had the advantage of allowing patients more time to think about their health and responses.

*“Normally I’d just do it beforehand or like, do you know, while I’m waiting for the Doctor to call me in after my bloods, I’d just complete it then. (Transplant patient 1)*

*“I think like I said maybe if you do it a couple of weeks before or something and then go through it when we’re there at the consultation and that’d probably be quite good…” (PSC patient 4)*

*“To get the most honesty out of people, do the questionnaires at the patients’ own comfort and their own space.” (Transplant patient 3)*

(ii) Access to patient data

All the patients wanted their consultant to have access to their PROM data and three patients went further to say they had no reservations about who had access to their ePROM data as long as it was for patient care, clinician training or research purposes.

*“….any problems I’d like the consultant at Queen Elizabeth Hospital [to know] they’re marvellous and they sound as though they care.” (PSC patient 2)*

*“I don’t really mind. I mean, if it helps in research or anything, then, you know, I’m all for it.” (PSC patient 3)*

(iii) Responding to ePROM data

Clinicians suggested that the severity of issues highlighted by ePROM data would determine the scale and speed of clinical response. Matters judged life threatening would be given priority and dealt with swiftly by the consultant while other issues might require nurses telephoning to patients to obtain more information and/or rescheduling clinic appointments to an earlier date.

*“But it depends what we’re identifying doesn’t it? If it’s, you know my tummy aches when I stand, that’s something that we can say we’ll look into it. If somebody declares they’re literally suicidal, I … we have no … realistic option but to deal with that there and then.” (MDT focus group participant)*

(iv) Patient-related issues

Participants mentioned the issue of patients with special needs or learning disabilities suggesting that a decision needs to be made about who provides proxy data for this groups of patients. It was recommended that questionnaires should be provided in other languages for patients who cannot read and write in English.

*“And then with someone with special needs, again you have to decide who is going to advocate for them because, I mean you want to get a true … opinion” (MDT focus group participant)*

*“And I guess it may be useful for those patients, for whom English is not their first language, to have some of these questions and there, available in their own language.” (PSC doctor 2)*

Patients becoming bored completing the same questionnaires over time and privacy issues were mentioned as issues that warranted careful consideration. A transplant patient, who is on immunosuppression therapy, raised the issue of hygiene if ipads were provided for use in clinic.

*“I wouldn’t mind but I don’t know, it might get a bit boring [laughs], you know what I mean?” (Transplant patient 5)*

*“I suppose, and obviously…..if … there’d be certain questions that I wouldn’t want somebody to fill in if they felt there was an observer.” (MDT focus participant)*

*“I have to be very aware of hygiene and stuff so if someone has just used that iPad with a cough or cold I would be very reluctant to use that one.” (Transplant patient 3)*

### Facilitators and barriers of implementation

Clinician enthusiasm was considered a facilitator for the implementation and use of ePROMs. The use of computer adaptive testing was suggested as a way to reduce patient burden and facilitate the use of ePROMs. It was also suggested that sending patients reminders would improve completion rates.

*“So I guess us being enthusiastic about it [laughs].” (PSC doctor 2)*

*“If you could reduce the size of the questionnaire, depending on their answers, so you know computer adaptive testing, that would be helpful.” (PSC doctor 5)*

*“I suppose the main barrier is that people have very good intentions to fill them out and then just forget…” (PSC doctor 6)*

A lack of awareness among patients and clinicians of the importance and potential benefits of collecting ePROMs was cited as a potential barrier. The time constraints during clinics could prevent clinicians from acting on ePROM results and this could become a barrier to the use of ePROMs. Patients’ computer literacy levels, language and access to internet or computer or phones were other potential barriers identified.

*“They won’t understand how it helps them. You might discover that clinicians don’t think it’s important, because they’ll say, well this is just academic, it’s just a way of measuring something that I know, it’s designed to find out more symptoms than are relevant, so they’ll just say, well I don’t think it’s important.” (PSC doctor 3)*

*“And when you need to stop the consultation for…you need half an hour consultation if somebody reveals that they’re feeling suicidal and you know in the middle of a big transplant clinic … I just think it needs to be thought out, you can’t just introduce it and then just sort of, it’s like an unexploded bomb then.” (MDT focus group participant)*

*“There will be a group who is not, who aren’t as comfortable, so it’s important to have the other option, as well as the electronic option.” (PSC doctor 5)*

## Discussion

Our study found that patients and clinicians supported the use of PROMs as part of patient management and identified a number of potential benefits. They evaluated selected questionnaires, identified various practical issues that need to be considered and outlined facilitators and barriers to the use of PROMs/ePROMs in routine practice.

Participants generally believed that the use of PROMs in routine clinical practice could be of significant benefit to patients and clinicians and may facilitate a more holistic, patient-centred, approach to care. Participants specifically proposed that the use of PROMs could improve patient-clinician communication as suggested by numerous studies [41, 42]. It is possible that by facilitating better communication between patients and clinicians, and improving patient engagement with their care, the use of PROMs may have a positive impact on treatment adherence [43, 44]. The use of PROMs could potentially assist with the management of patients transitioning from paediatric to adult care services by promoting their engagement with services and providing healthcare providers an avenue to assess and monitor patients’ adjustment to the changes occurring during this process.

Other potential benefits identified by the participants and supported by literature were: improving the monitoring and reporting of symptoms; highlighting issues to address during clinical consultations; aid patient recall of issues to discuss in clinic and management of appointments [45, 46]. It should be noted that although patients in our study supported the use of PROMs in their future management, a number of them felt that PROMs and/or virtual clinics should not replace regular face-to-face clinical consultations. At the moment, high quality evidence to support the use of virtual clinics is limited and equivocal [47–49].

Participants felt the disease-specific questionnaires (CLDQ and PedsQL-TM) were more relevant and more useful than the generic SF-12 and EQ5D. Studies have suggested that generic measures like the SF-12 and the EQ5D may be inadequate for assessing the impact of rare diseases on patients and recommend using disease-specific measures when available [50, 51]. Although the CLDQ has been reported to have good test-retest reliability and cross-sectional validity, [52] this should be interpreted with caution as the evidence was obtained from studies that combined the results of patients with PSC with that of patients with primary biliary cirrhosis (PBC) and hepatitis B & C [53, 54]. Preliminary validation of the PSC PRO, a new measure developed in accordance with FDA guidelines suggests good psychometric properties [55, 56]. The PedsQL-TM has been reported to have good reliability and validity in young patients with transplant [34, 35].

While patients and clinicians in our study expressed the desire for questionnaires with greater disease specificity, the excessive variations that exist within many rare conditions make the development of such measures impractical. Rüther et al. reported a similar situation in their study of patients with lysosomal storage diseases [50]. The comments from the patients and clinicians have also highlighted the important aspects of PRO development and administration that may require clarification during the design and implementation of an ePROM system.

Clinicians raised the issue of delegating the completion of PROMs to carers of patients with learning disabilities. Whilst this might seem a logical solution in this group of patients, research has shown that the use of proxy respondents may lead to response bias with proxies reporting more impairment in affect, physical and cognitive function [57]. Furthermore, the European Medicines Agency (EMA) and the US Food and Drug Administration (FDA) discourage the use of proxy data [5, 58].

Our study participants suggested adding comment boxes to the questionnaires. While comment boxes might help individualise questionnaires and allow patients the opportunity to provide additional information, they may be difficult to process into usable information in routine clinical practice as manual mapping may be time consuming [59]. Chung et al. found that 62% of patients’ free text electronic entries could be structured into drop down menus options using computer programming [59]. The use of Computer Adaptive Testing (CAT), although in its infancy, may simultaneously reduce questionnaire burden and tailor questionnaires to the specific needs of the individual [4].

Participants believed clinician interest would be a key factor for successful implementation of ePROMs while a lack of awareness among patients and clinicians, time constraints, patients’ literacy levels and access to internet/electronic devices were identified as potential barriers to the use of PROMs. These findings are in keeping with the findings presented in a number of previous studies [46, 60].

One of limitations of this study was the difficulty of recruiting a sufficient number of participants to ensure that data saturation was achieved. However, this is not unique to our study as other studies involving patients with rare diseases have encountered similar difficulties [39, 61, 62]. The small sample sizes makes it difficult to preserve the anonymity of participants hence the decision not to provide a detailed socio-demographic description of the participants [63]. In addition, the transferability of some of our findings may be limited to settings that manage patients that closely match our study participants in terms of disease and age.

## Conclusion

The use of PROMs/ePROMs in the management of patients with rare diseases may facilitate patient-centred care by promoting patient-clinician communication, highlighting aspects of HRQOL that are important to patients and encouraging patient involvement in their care. The insights from this study could inform the design, development and implementation of an ePROM system for patients with rare diseases.

## Supplementary information

**Additional file 1:** Primary Sclerosing Cholangitis Patient Interview Topic Guide.

**Additional file 2:** Primary Sclerosing Cholangitis Participants Interview Topic Guide (Clinicians).

**Additional file 3:** Patient information sheet for renal transplant recipients.

**Additional file 4: Table S1.** Potential benefits of PROMs and views on selected questionnaires.

**Additional file 5: Table S2.** Views of selected questionnaires.

**Additional file 6: Table S3.** Practical considerations.

**Additional file 7: Table S4.** Facilitators and Barriers.

## Abbreviations

CAT: Computer adaptive testing
CLDQ: Chronic Liver Disease Questionnaire
ePROMS: Electronic PROMs
EURORDIS: European Organisation for Rare Diseases
EQ-5D: EuroQOL-5 dimensions
HRQOL: Health-related quality of life
MDT: Multidisciplinary team
NHS: National Health Service
PedsQL: Paediatric quality of life inventory
PRO: Patient-reported outcome
PROMs: Patient reported outcome measures
PSC: Primary sclerosing cholangitis
QOL: Quality of life
SF-12: 12-item short form health survey
UK: United Kingdom
