# Supplementary material for: Patient and clinician opinions of patient reported outcome measures (PROMs) in the management of patients with rare diseases: a qualitative study
Source: Health Qual Life Outcomes. 2020 Jun 10;18:177. doi: 10.1186/s12955-020-01438-5 (PMC7288678; doi:10.1186/s12955-020-01438-5)
Supplement: Supplementary file 1 — Additional file 1: Primary Sclerosing Cholangitis Patient Interview Topic Guide. [file 12955_2020_1438_MOESM1_ESM.docx]

**Primary Sclerosing Cholangitis Patient Interview Topic Guide**

Remember I posted two questionnaires (PROMs) to you. The two PROMs are short form- 6D [SF-6D] and chronic liver disease questionnaire [CLDQ]) and were developed by our colleagues to measure the impact of disease the quality of life (QOL) of patients. I will give you few minutes to look over these questionnaires again, and I will ask you about your opinion about the PROMs.

1) What is your first impression about the questionnaires?

**Probes include**

What do you mean by….?

You said ….

Can you tell me more about ….?

Could you explain a little more …?

You mentioned …. Is there anything else you’d like to add?

2) If these questionnaires are used in the clinic, how often would like to complete these PROMs, what format would be best for you?

3) If you have any problems how do you want these to be addressed?

4) What you think are the barriers to the use of electronic PROMs /questionnaires

5) How do you like the clinicians to get in touch with you about your response to the questions/questionnaires?

6) Who would like to have access to your response to the questionnaires?

7) How often do you go for follow up clinics?

8) If the answer to the questionnaires show’s that you are doing fine are you willing to space out (have less frequent) clinic appointments or use virtual clinic?

9) Looking at the two questionnaires is there any aspect/part of your disease that is not covered in these PROMs?

10) I’d like to give you the opportunity to say anything else about the questionnaires, your experience of completing them or anything else we discussed.
