# Supplementary material for: Patient and clinician opinions of patient reported outcome measures (PROMs) in the management of patients with rare diseases: a qualitative study
Source: Health Qual Life Outcomes. 2020 Jun 10;18:177. doi: 10.1186/s12955-020-01438-5 (PMC7288678; doi:10.1186/s12955-020-01438-5)
Supplement: Supplementary file 2 — Additional file 2: Primary Sclerosing Cholangitis Participants Interview Topic Guide (Clinicians). [file 12955_2020_1438_MOESM2_ESM.docx]

**Primary Sclerosing Cholangitis Participants Interview Topic Guide (Clinicians)**

Remember I gave you two questionnaires (PROMs) to read through after the last liver clinic. The two PROMs are short form-12D [SF-12D] and chronic liver disease questionnaire [CLDQ].

I should say at this point, there are no right or wrong answers. I am looking for your opinions and thoughts, whatever they may be.

1) If you were to use these questionnaires in the clinic, what format do you think will be best for your patients and why?

2) If you were to use these questionnaires in clinic, how often would you like the patients to complete these PROMs and why? One suggestion is to embed in myHealth patient portal…

3) How would you like to use the results or information from any PROM? Which of the result/information will be important to you and why?

4) How would you use the findings of the information obtained from the PROMs and why?

**Probes include**

What do you mean by….?

You said ….

Can you tell me more about ….?

Could you explain a little more …?

You mentioned …. Is there anything else you’d like to add?

5) If a patient has any problem when completing the PROM how would you address it in the clinic?

6) What do you think might be the barriers to the use of PROMs particularly (electronic PROMs)?

7) What would help to facilitate the use of electronic PROM?

8) Thinking specifically about these questionnaires, do you feel they capture things relevant to the patients?

9) Looking at the two questionnaires is there any aspect/part of the patient’s condition that is not covered in these PROMs and why?

10) I’d like to give you the opportunity to say anything else about the questionnaires or anything else we discussed.
