# Supplementary material for: Patient and clinician opinions of patient reported outcome measures (PROMs) in the management of patients with rare diseases: a qualitative study
Source: Health Qual Life Outcomes. 2020 Jun 10;18:177. doi: 10.1186/s12955-020-01438-5 (PMC7288678; doi:10.1186/s12955-020-01438-5)
Supplement: Supplementary file 3 — Additional file 3: Patient information sheet for renal transplant recipients. [file 12955_2020_1438_MOESM3_ESM.doc]

**PARTICIPANT INFORMATION SHEET**

**TITLE OF RESEARCH STUDY: PATIENT REPORTED OUTCOME ASSESSMENT IN RENAL TRANSPLANT**

We would like to invite you to take part in our research study. Before you decide whether you want to take part in this study it is important you understand why the research is being done and what we would be asking you to do. Please take time to read the following information carefully and feel free to discuss or talk to anyone about this research. Contact us if there is anything that is not clear or if you would like more information about the study (see contact details at the end of this form).

We would like you to take your time to decide whether or not you wish to take part in this research study. If you are interested in taking part in our research, please return the attached slip to the researcher, who will contact you to answer any questions you may have and will discuss your involvement in the study.

**INFORMATION ABOUT THE STUDY**

This information sheet consists of two parts: Part 1 tells you the purpose of this study and methods. Parts 2 gives you more detailed information about the conduct of the study.

**PART ONE**

**WHAT IS THE PURPOSE OF THE STUDY?**

Previous research suggests that coping with renal transplant and transitioning from paediatric to adult –focused care can be challenging due to poor outcomes. One way to understand why adolescent have poorer outcomes is through patient reported outcomes. Patient-Reported Outcome Measures (PROMs) are questionnaires that collect information directly from patients about how they feel or function. This information may include signs and symptoms, perceptions or feelings, well-being, quality of life, treatment satisfaction or benefits and side effects of treatment. This study will help clinicians to understand why adolescent have poorer outcomes than other age banded groups. In addition, the study will help identify appropriate measures used for renal transplant patients and to determine how best to capture data that is meaningful to inform patient care and also acceptable and helpful to patients. We would like to gain your opinion on the use of PROMs in the NHS.

**WHY HAVE I BEEN INVITED?**

You are invited to participate in this research study because you have been referred to us from the clinical team as a person between the ages of 16-25 years who is transitioning following renal transplant. We need to conduct the study involving people between the age of 16-25 years who are transitioning following renal transplant in order to address our research questions.

**DO I HAVE TO TAKE PART?**

Participating in this research is completely voluntary. It is up to you to decide whether or not to take part in the study. If you decide to take part we will ask you to sign a consent form.

If you decide not to take part or withdraw at a later time, your medical care will not be affected in anyway.

**WHAT WILL HAPPEN TO ME IF I TAKE PART?**

If you decide to take part in the research project, you will be interviewed by a researcher who will ask you questions about your experience living with renal transplantation. The interview will also explore your view about using PROMs in the NHS. We will provide you with an example questionnaire and ask you to share your thoughts on a) how helpful the questionnaire might be b) how often you may be willing to complete such a questionnaire c) whether you would prefer to complete the questionnaire on paper or electronically.

You will be invited to take part in an interview, which is expected to last between 45 to 90 minutes. The interview will be conducted in a place of your choice that is convenient for you, for example, the University of Birmingham, the Queen Elizabeth hospital or via the phone. The lead researcher will contact you to arrange the interview and will ask you if you would prefer to conduct the interview in one go, or to split the interview into smaller sessions. They will also ask if there is anything that can be done to ensure your comfort during the interview (for example, scheduling regular breaks during the interview session).

The interview will be recorded on an audiotape and typed up on a computer. The audio tapes will be stored in a secure locked place at all times, and the computer is protected in accordance with the Information Security Policy of the University of Birmingham and Data Protection Act Regulations.

If you are interested, the preliminary and final results will be sent to you.

**EXPENSES AND PAYMENTS**

We will give you £10 (cash or voucher) to cover reimbursement for your travel expenses/time.

**WHAT WILL I HAVE TO DO?**

You will be asked to consent to taking part in the research project by signing the consent form.

On completion of the consent form, you will take part in one 45-90 minutes interview.

You will be asked to read and comment on (if appropriate) a summary of preliminary results.

**WHAT ARE THE POSSIBLE BENEFITS OF TAKING PART?**

There are no direct medical benefits to you; however, you may find it beneficial to discuss your feelings and experience of transitioning following renal transplant. Also, the information obtained from yours and the other interviews we conduct will help health professionals gain a better understanding of how transitioning following renal transplant impacts upon patient’s lives.

**WHAT ARE THE POSSIBLE DISADVANTAGES AND RISKS OF TAKING PART?**

When talking about what it is like transitioning following renal transplant, it is possible you may experience distress during the interview. If this happens you will be asked if you wish to delay or discontinue the interview. The interviewer will try to deal as sensitive as possible with any problems that might arise during the interview and will be able to advice on mechanisms for support. If the interviewer has concerns about the welfare of a participant, they will first discuss the issue with the supervisory team and if indicated, they may encourage the participant to consult the clinician involved in their care.

**WHAT WILL HAPPEN TO THE RESULTS OF THE RESEARCH STUDY OR WHEN THE RESEARCH STOPS?**

We aim to publish the findings of the study in scientific journals. You will not be identified in any report or publication. If you wish, we will send you a summary of the results once the study is completed.

***IF THE INFORMATION IN PART 1 HAS INTERESTED YOU AND YOU ARE CONSIDERING PARTICIPATION, PLEASE READ THE ADDITIONAL INFORMATION IN PART 2 BEFORE MAKING ANY DECISION. THANK YOU FOR TAKING THE TIME TO READ THIS INFORMATION***

**PART TWO**

**WHAT WILL HAPPEN IF I DON’T WANT TO CARRY ON WITH THE STUDY?**

You are free to withdraw from the study at any point prior to my data being integrated into data set (1 week days following the interview), without giving any reason.

**WILL MY TAKING PART IN THIS STUDY BE KEPT CONFIDENTIAL?**

Yes. We will ensure that all information collected about you during the course of the research will be kept strictly confidential. Any information will have your name and address removed and a code number substituted so that you cannot be recognised by it. The records used for analysis will only have this code number on it. Audio tapes of the interview will be used to make a typed copy after which we will destroy the original recording. The typed copy will then be anonymised. A professional transcription service will assist in the transcription of the interviews. The transcriber will sign a Confidentiality Agreement and will comply with the data protection act. All typed copies will be destroyed after 10 years in line with Data Protection Act Regulations. We will only use anonymised quotes from the interview in the study publication. The researcher may be required or compelled to disclose information obtained during in the interview if they become concerned about your wellbeing or safety, and may have a duty to report to the appropriate research supervisor.

**WHO IS ORGANISING AND FUNDING THE RESEARCH?**

The University of Birmingham is organising the research. The study is being funded by Metchley Park Medical Society.

**WHO HAS REVIEWED THE STUDY?**

The study has been approved by NHS Research Ethics Committee on (20^th^ May 2016). Approval number (16/WM/0198)

**FURTHER INFORMATION AND CONTACT DETAILS**

If you have any questions about any aspect of the study, you should speak to:

**FOR CLINICAL CONCERNS CONTACT:**

**SUPERVISORY TEAM:**
