# Supplementary material for: Patient and clinician opinions of patient reported outcome measures (PROMs) in the management of patients with rare diseases: a qualitative study
Source: Health Qual Life Outcomes. 2020 Jun 10;18:177. doi: 10.1186/s12955-020-01438-5 (PMC7288678; doi:10.1186/s12955-020-01438-5)
Supplement: Supplementary file 4 — Additional file 4: Table S1. Potential benefits of PROMs and views on selected questionnaires. [file 12955_2020_1438_MOESM4_ESM.docx]

| **Table S1. Potential benefits of PROMs and views on selected questionnaires** | | |
| --- | --- | --- |
|  | **Codes** | **Quotes** |
| **Potential settings** |  |  |
|  | As part of face-to-face clinical consultation | *“The questionnaires should be used so you review the patient on clinic days” (Renal transplant patient)* |
|  | Patient review outside clinic appointments | *“….We have mornings where we go through blood tests, pathology etc for every patient and assess any information added on to the records. I am assuming the clinicians have that sort of thing. Put these in the notes and compare with the notes. And if it is important information, get back to the patient saying ‘You are experiencing this maybe that’s your symptoms progressing….If it is not applicable, probably still ring the patient back, you know saying Dr so and so looked through your questionnaire and there is nothing to worry about etc etc Have done anything about it? It may be side effects. It could be other reasons for the problems. Is it being treated by the GP or not?” (PSC patient 1)* |
| **Potential uses** |  |  |
|  | As a conversation starter | *“About the questionnaires in general … I mean it is, as x said, it’s an introduction. So in terms of its usefulness in routine practice, then yes I think it could be, but as a, as a, it’s not the end result, it’s something that’s helpful.…” (PSC doctor)* |
|  | To improve patient-clinician communication | *“I think that would be a valuable addition to the care that we provide in terms of improving, yeah, improving communication between patients and their clinician.” (PSC doctor)*  *“But that’s really where PROs come in. It’s more about patients don’t always tell doctors their symptoms, unless they’re asked, and even if they’re asked, they may not report them, whereas with a PRO of course, if they fill it in, then they have, not have to, but they fill in all the questions, don’t they? So you get much better sense. And we know that from other liver diseases that we work in, so we work in the liver disease called PBC, and we know that patients will under report to their doctor verbally their symptoms, but if you quantify their symptoms you’ll get a higher response rate.” (PSC doctor)* |
|  | For better reporting of symptoms | *“But that’s really where PROs come in. It’s more about patients don’t always tell doctors their symptoms, unless they’re asked, and even if they’re asked, they may not report them, whereas with a PRO of course, if they fill it in, then they have, not have to, but they fill in all the questions, don’t they?” (PSC doctor)*  *“Sometimes it does pick up stuff that you wouldn’t otherwise … know about or see about. I think people are sometimes, I understand what you’re saying, sometimes they won’t at all, but sometimes….” (MDT FG participant)* |
|  | Holistic approach to care | *“I think a way of just making sure that things aren’t being missed and that you’re looking at the patient maybe more holistically” (PSC doctor)* |
|  | Highlight issues for review during clinical consultation | *“I think they’re good to highlight things which you wouldn’t otherwise pick up on in consultation, er, because there’s often so much to kind of go through, I think they pick up the stuff that generally clinicians are really not as good at asking about.” (PSC doctor)*  *“Like I would look at how the patient walked into clinic, to see whether they were having difficulty walking or if they looked like they were in pain when they were walking, so I wouldn’t necessarily need a tick box to tell me, it would … it would be useful though to explore that and find out exactly what was going on.” (PSC doctor)* |
|  | Monitoring   - Changes in condition - Between clinic appointments - Response to treatment - Side effects of treatment | *“I do believe from just anecdotal sort of experience, that there are certain symptoms which may predict deterioration before we see changes in blood tests or before we see a sudden deterioration clinically” (PSC doctor)*  *“Serially it may be more useful if people feel that you know it’s a repeated measure and they can communicate how things actually change. I think many of the questions are very helpful” (PSC doctor)*  *“Yeah, I come in every year anyway so I’d be good to do it every year and then you can see what happened last year to the next year can’t you, if it’s getting worse or better.” (PSC patient 4)*  *“And I guess if there’s a way of getting them to fill these in electronically, even though they’re not coming to clinic that would be a way of reassuring ourselves that even though we haven’t seen them, they’re still fine.” (PSC doctor)*  *“I suppose it depends on what you were trying to measure, because if you were trying to measure a difference in the, in how the patient thoughts their outcomes were, dependent on what treatment or care that you provided to them, you’d possibly want to do that quite regularly to try and monitor if there was a change and hopefully an improvement.” (PSC doctor)*  *“I think is, is really important because although we say you’ve got lovely kidney function, you look very well, etc.” (MDT FG participant)*  *“We have encounters with the clinic you really like and some your aren’t, you might be on new medication that you weren’t on before and you had experience of that medication so you kind of like think about it but it just depends on how often you do it” (Renal transplant patient)* |
|  | Spacing clinical appointments | *“Q: if the answers to the questionnaires show that you are doing fine, are you willing to space your appointments or use virtual clinic?*  *Yeah, I guess. As long as I had access to it when I had problems, yeah.” (PSC patient 4)*  *“Yeah, I’m happy to do it, yes.” (PSC patient 3)*  *“Well, er, no, because I think that according to the consultant, er, I, we need to, to have a visit and be seen once a year. Er, and that, those are his instructions. We, we go on the, on the request of the consultant, don’t we, ‘cos we’re totally in his hands? And if the questions here could be slightly ambiguous or not complete, then that could determine, er, how often we see, we’re seen by the consultant and he knows exactly the questions to ask, whereas the form perhaps doesn’t ask all the questions.” (PSC patient 2)* |
|  | Facilitation of multidisciplinary team (MDT) approach | *“It might be a way of getting other allied health professionals on board and do a one stop service instead of a fragmented service, like what we do now. So if they need to see a dietician, the dietician’s there, the physiotherapist is there, the pharmacist is there, you know the kind of … the psychologist is there, the nurse is there, the doctors are there, so it’s a one stop shop, so that if they have problems pertaining to any of this, that they can get advice from whoever is in the clinic.” (PSC doctor 4)* |
|  | Facilitate research | “I’d prefer to do questionnaires to help researchers find out more. So I’m happy to do them and I think, I think everyone else would be as well” (Renal transplant patient) |
