# Supplementary material for: Patient and clinician opinions of patient reported outcome measures (PROMs) in the management of patients with rare diseases: a qualitative study
Source: Health Qual Life Outcomes. 2020 Jun 10;18:177. doi: 10.1186/s12955-020-01438-5 (PMC7288678; doi:10.1186/s12955-020-01438-5)
Supplement: Supplementary file 5 — Additional file 5: Table S2. Views of selected questionnaires. [file 12955_2020_1438_MOESM5_ESM.docx]

| **Table S2. Views of selected questionnaires** | | |
| --- | --- | --- |
| Chronic Liver Disease Questionnaire (CLDQ) | - Quite good - Quite relevant - Most symptoms covered - Fine - Illness could be due to other causes (attribution issues) - Accurate reflection | *“I think, I think one of them is good, the one that's got the 30 questions on. I think that the other one is just like, I don’t know, I didn’t think, I don’t know, I didn’t really… The, the first one, er, the, like the one that’s got the 30 question, the multiple‑page one.” (PSC patient 4)*  *“Well, before my surgery, possibly quite a lot of things, you know, would have, sort of, you know, erm… Well, most of these things did actually apply to me, so…” (PSC patient 3)*  *“No, it’s, it’s absolutely fine. There’s no problem there. Erm, if there was anything that was seriously wrong that I, I picked up on, er, then obviously I could, I could, you know, sort of give you the answers to it, but when there doesn’t seem to be anything wrong with me at the moment regarding PSC it’s difficult to answer the question.” (PSC patient 2)*  *“Yeah. Oh no, the form’s fine, yeah. It’s just that it relates to the last four weeks and if there is something else wrong with your health, perhaps a bug or a cold or flu or something like that, er, then, er, then you’re, you’re bound to feel a little bit, er, er, a little bit off...” (PSC patient 2)*  *“But, in terms of looking at all those activities etc., etc., then I would say yeah, most of them do apply to my condition. So, from that point of view, I would say it’s, kind of, quite accurate, it reflects the PSC condition. (PSC patient 1)*  *I suppose it depends. Everyone has different symptoms, don’t they? But the symptoms that I’ve experienced so far, I think the majority of them are covered on here I think personally.” (PSC patient 1)* |
|  | Suggestions for improvement   - Comment box - Need for tailoring to individual needs - Direct questions on fear | *“Er, I think there ought to be a box to put comments in.” (PSC patient 2)*  *“Maybe there should be a box there saying, ‘is there any other conditions or any other factors that affect you?’ This, this, this, this, but there’s nothing to say ‘do you have any other comments or is there any other condition or any other symptoms?’ So, you’re kind of like, generally.” (PSC patient 1)* |
| SF12 | - Not PSC-specific - Not personally relevant | *“Er, well, yes, er, because it asks generally how your health is, er, but it doesn’t, but your health problems vary from day to day with, er, other, oh, I don’t know, causes...” (PSC patient 2)*  *“That’s not kind of, and none of that is relevant to me so.... maybe to other people it might be but… but the first one that, that’s just the one-page one, I don’t think it does.” (PSC patient 4)* |
|  | | |
| Paediatric quality of life inventory (PedsQL) | - Relevant and helpful - Really good | *“But this thing here about the transplant stuff, about the medicines and how they make you look and feel and how people treat you differently and … I think that’s very relevant to us, I think that’s the information that we’re kind of talking about in clinics quite often.” I think many of the questions are very helpful.” (MDT participant)*  *“No I don’t think they’re relevant to me because it’s been such, it’s been so long after my transplant” (Renal transplant patient 3)* |
|  | Suggestions for improvement   - Questions about fear and unmet needs - Question for family views of transplant - Question on experience of transplant process - Questionnaire for parents | *“I mean things like what do your family think about your transplant and do you think that they are happy for you or do you think that they are not like some patients they’re not allowed to take transplants, other religions they do allow you to so there should be a little, I know it’s a bit taboo but there should be a question to see if they are comfortable with having one or not.” (Renal transplant patient)*  *“Maybe one, like, sort of, like, how comfortable did you feel, like, during the process of transplant and all that and how, how well did you feel you were looking after and maybe a question like that with a scale of 1 to 10 maybe ‘cause me, for me, it was fantastic, like, you know.” (Renal transplant patient)* |
