# Supplementary material for: Patient and clinician opinions of patient reported outcome measures (PROMs) in the management of patients with rare diseases: a qualitative study
Source: Health Qual Life Outcomes. 2020 Jun 10;18:177. doi: 10.1186/s12955-020-01438-5 (PMC7288678; doi:10.1186/s12955-020-01438-5)
Supplement: Supplementary file 6 — Additional file 6: Table S3. Practical considerations. [file 12955_2020_1438_MOESM6_ESM.docx]

| **Table S3. Practical considerations** | | |
| --- | --- | --- |
| **Categories** | **Codes** | **Quotes** |
| **Practical considerations** | | |
| Availability of resources |  | *“I think to sit down with something this length and do it electronically in a clinic would be harder because you’d have to have let’s say like an iPad or something and you could only really have one person doing it at a time unless you had a clinic that had ten, you’re not going to have maybe that resource to do that many at the same time.” (PSC doctor)*  *“The other thing about these is that you do have to have a capacity in the clinics to do something about them because (small laugh) from the other point of view, I’ve been asked these and actually it’s very disheartening if you’re asked to fill this in and then nobody does anything about it.” (MDT participant)* |
| Optimal time for collection | In the waiting room | *“The times where this information is most likely to be captured is usually just in the waiting room whilst they’re waiting for their delayed clinic appointment, because they’ve got nothing to do at that point, and afterwards, because the appointments often run late, they will sort of probably want to go and start their day.” (PSC doctor)*  *“Just the practicality of getting hold of the patients, getting, giving them the iPad)…” (MDT participant)*  *“… getting them to do it when you know we’re (small laugh), we’re all trying to get them into our clinic room or give them, show them to the nurses, or X trying to get them … and it’s quite busy, you know, there’s quite a lot going on in the clinic, it would just have to be quite organised about how to do it.” (MDT participant)*  *“Erm, I don’t know. Maybe if I complete it at the clinic then I can take it with me to my consultation and maybe drop it in. Yeah. Yeah, I mean I could do it when I was in the waiting room. That’s probably not a bad idea. Probably occupy people but like I said some people might not be technically, if it was on a paper then it might be all right at the waiting room.” (PSC patient 4)*  *“Normally I’d just do it beforehand or like, do you know, while I’m waiting for the Doctor to call me in after my bloods, I’d just complete it then. (Transplant patient 1)* |
|  | Prior to clinic appointments | *“That’s why if you do it through My Health, it’s in their own environment, they haven’t had the stress of coming to the hospital, the clinic being late, nowhere to sit, hard to park the car, which is not their PSC at all is it?” (PSC doctor)*  *“It sort of, it sort of depends on how, how much you’d get them to complete it before clinic, I suspect it would be very low, I don’t think you’d get very many completed before clinic.” (MDT participant)*  *“So they largely won’t know their My Health log-ins, even though we have set them up. So I think you’d be better off, if we were trying to do a PROM in our clinics, like this, like pre-clinic.” (MDT participant)*  *“This questionnaire is good. It kind of jogs your memory. Maybe you could fill this in before the clinic. It might also be done quickly in clinic. But doing it in advance will give them more time to think about their answers. But yes something like that will be brilliant. It covers most things.” (PSC patient 1)*  *“Probably the best, the best time to do it is probably a couple of days before the appointment to be honest with you. Maybe you can email it on a website or something and somebody just goes in and fills it in and then when you’re in your consultation you can, the clinicians can go through it with you.. That might be a good, better way of doing it maybe.” (PSC patient 4)*  *“I think like I said maybe, you know, maybe if you do it before, a couple of weeks before or something and then get it to, go through it when we’re there at the consultation and that’d probably be quite good…” (PSC patient 4)*  *“To get the most honesty out of people, do the questionnaires at the patients’ own comfort and their own space.” (Transplant patient 3)*  *“I wouldn’t mind doing that then I could do it on my own at home with a cup of coffee.” (Transplant patient)* |
| Frequency of collection | Yearly  Dependent on how quickly symptoms change  Every six months  Whenever one is sent | *“I mean I guess it’s something you might think about doing on a six monthly or on an annual basis if you’re … So I mean often we see these patients either every six months or once a year, and I think it would be useful to have something like this, then you could sort of document how things have changed over the last year” (PSC doctor)*  *“I think in terms of trying to capture symptoms I think it’s good to do it every time because at least that way you kind of pick up you know what’s going on, what might have changed and things that you need to talk about in consultation, it gives, I think if patients get it before they come in to see you then it also prompts them a little bit to think about something that they might want to say to you.” (PSC doctor)*  *“Every three months, if they were coming to clinic, so in between you may not necessarily want them to complete every time.” (MDT participant)*  *“Probably, probably, probably every year or something maybe… Yeah, I come in every year anyway so I’d be good to do it every year and then you can see what happened last year to the next year can’t you, if it’s getting worse or better.” (PSC patient 4)*  *“Oh. Well, er, perhaps erm, I don’t, the problem is that I don’t know how quickly symptoms can change on PSC. Er, so the answer to that is not easy, simply because, erm, I could be okay one day and two months later there could be a change. So, er, I mean, you would have to tell me how quickly things would change generally, I suppose. I mean, would you say it was six months?” (PSC patient 2)*  *“I will do. Yeah whenever. Whenever I have a questionnaire.* *I would definitely provide it to them if it would definitely help.” (PSC patient 1)*  *“I used to go every three months but now like, touch wood my health is really good ,I go every six months, so I wouldn’t mind filling one in to be honest, yeah, I’d fill it in like every six months or every three months” (Transplant patient)* |
| Mode of administration | Electronic vs paper vs face-to-face chat | *“Well I’d like to see them all on iPads nowadays…You know you can get them to do the questionnaires more can’t you?” (PSC doctor 3)*  *“If you really want it, it needs to be on the phone, I think it’s very quick isn’t it? Everyone’s got their phone.” (PSC doctor)*  *“I think they’re a lot more engaging on an iPad, yeah. Why would that be better on an iPad? I just think they’re a bit more … I honestly think they hate pen and paper” (MDT FG participant)*  *“I’d be concerned if you gave that to them as a bit of paper in a waiting room that it would just get left on the seat and people wouldn’t fill it on.” (PSC doctor)*  *“Even when they were waiting, bored, in our clinic and I’d start you know doing the, for them to fill out the questionnaire about transition, they just tick box and then leave it there and not even, they weren’t even reading it, they were just ticking boxes …” (Transplant doctor)*  *“Either. It doesn’t matter to me to be honest, yeah. I understand some people might want it on paper because they don’t like it electronic.” (PSC patient 4)*  *“Oh, I don’t know. I always prefer it electronically.” (PSC patient 3)*  *“I don’t mind paper, but I would prefer electronically. You just log on and it’s much quicker that way, isn’t it? That’s my personal view, anyway.” (PSC patient 3)*  *“Personally, I think it’s very good. First of all, it’s environmentally-friendly as well, so, you know, not using the paper and, er, and – it’s also much better to keep the record electronically, isn’t it? See, when I worked, I worked for a company and they already could – well, they were doing everything electronically and that’s why it’s… It’s, er, less hassle, it’s much quicker, it’s more efficient, more accurate…” (PSC patient 3)*  *“I guess it’d be paper really.” (PSC patient 2)*  *“I, I’ve got My Health, I’ve, but I’ve never used it ‘cause I keep forgetting my password for it.” (Transplant patient)*  *“Erm…[Laughter] Er, well, er, I prefer a general chat, to be honest. Erm, rather an electronic questionnaire, but the electronic questionnaire gives you time to think about the, the answer, er, so yes, yes, I think it is a good idea. But not everybody has a computer.” (PSC patient 2)*  *“With electronic you have to mess about. I hate doing things electronic. You have to tick here or there. But electronic means you can access it straight away. From that point of view, it’s quite good. If you are busy like me trying to find paper here and everywhere is a nightmare. So electronically is normally the best way forward. It is just filling it in electronically takes more time.” (PSC patient 1)*  *“I think paper based, yeah…” (Transplant patient 1)*  *“I felt like it was better because then I could write down as much as I felt like, yeah, yeah” (Transplant patient)*  *“I feel like it would be good online as well because like if you don’t go to the clinic or you can’t make it to the clinic you can submit it online and you can still get your answers that way as well” (Transplant patient)*  *“If you just, if you just like gave us an, an iPad and just go, that’ll be fine.” (Transplant patient)* |
| Choice of platform | Preference for MyHealth | *“I think it would be good to be on – on MyHealth, er, because it’s a potential way for people to access – access it, er, it does mean yeah a little bit more time which they get to fill it in as well.” (PSC doctor)*  *“So we could do it through MyHealth, it’s just a bit of a faff for us to do this in the clinic because the patient has to have set up their MyHealth account and then remember their log-in.” (MDT FG participant)*  *“Sorry, going back to MyHealth as well, it’s, it’s the majority of the time it’s the parents that are looking at MyHealth.” (MDT participant)* |
|  | Interest in MyHealth | *“I can actively access it then, and see it whenever I want to? I would be interested in that, yes.” (PSC patient 3)*  *“I tend not to look at it too much. Initially, My Health, I thought was good. Erm, it was accessible and then you could do this, that and the other. But then I realised it had got a few restrictions. I didn’t really go onto the help groups, etc., that kind of thing.” (PSC patient 1)* |
| Access to patient responses | Consultant  Anyone on the medical/research team  Anyone if it will be of benefit | *“Probably the consultation, er, the, the, the consultant* *really I guess.” (PSC patient 4)*  *“I don’t really mind. I mean, if it helps in research or anything, then, you know, I’m all for it.” (PSC patient 3)*  *“Er, but yes, any problems I’d like, er, I would like, erm, the consultant at, erm, Birmingham Univer, erm, Birmingham Hospital there, Queen Elizabeth Hospital ‘cos they, they’re marvellous and they sound as though they care.” (PSC patient 2)*  *“Me, personally I’ve got no issues, but anybody who would get benefit from that. Even if it is not me, even if it’s the psychologist or doctor who gets benefit from that. For me, that’s a good thing. If there’s anything that can get you medically better, treatment-wise or diagnosis-wise, or prognosis, etc., etc., then for me that’s a good thing. For me, I would have no issues sharing it with anybody. I know, at the minute there’s issue with who can access records etc., etc., which I think is a shame. Because one of the things I am doing at the moment is talking about sharing records between community pharmacies and hospitals and doctors – at the moment they can’t. Pharmacists can only access primary care records only. They can’t access any other clinical details, for example, which is a shame, because sometimes, like with mental health. Yeah, so for me, it’s not a problem at all. I think it is a good think which could help to improve the patient outcomes and treatment options, etc., etc.” (PSC patient 1)*  *“Anyone that could possibly progress their own careers or any doctors that are in training that need this sort of information to help them progress or any research students like yourself that need this sort of information.” (Transplant patient)* |
|  | Anonymity | *“The thing is, in this case, you’ve got the patient details on, so that’s fine, it’s not anonymous, the patient’s details are on. Sometimes they are anonymous if the patient doesn’t put the details on. Then you will struggle to get a response.” (PSC patient 1)* |
| Responding to PRO alerts or results | Seriousness of alert to be considered | *“And if they are unhappy with any particular one … if there is a particular thing that stands out in the symptom profile, or in the patient’s disease understanding, then you know, then certainly sort of I get, go back to the individual and ask them” (PSC doctor)*  *“But it depends what we’re identifying doesn’t it? If it’s, you know my tummy aches when I stand, that’s something that we can say we’ll look into it. If somebody declares they’re literally suicidal, I … we have no … realistic option but to deal with that there and then. Not least because we’d feel very bad if a week later … we didn’t, they didn’t come back!” (MDT participant)* |
|  | Mode of response   - Email - Telephone - Letters | *“If there is a point later on or if something happens before their next clinic there’s been a change, then I respond to it in real time, so if they communicate with me, the first opportunity I have to reply back to them, I’ll try and reply back. I mean usually e-mail works pretty well, so …!” (PSC doctor)*  *“Probably I’d phone up or ask one of the nurses to have to phone them up, to try and clarify what’s changed and to see whether that’s something that might … I mean it might be some … so it depends a little bit what it was, it might be something that would, would trigger me to think, well we need to see them in clinic sooner, and just bring, to bring their appointment forward so we can see them sooner.” (PSC doctor)*  *“Preferably email” (PSC patient 3)*  *“Er, by, er, well, either by email or by telephone; I don’t mind. Erm, but if there are any questions I would imagine by email would be fine, yes.” (PSC patient 2)*  *“I would hope that whoever reads the questionnaire will read it and say ‘this concern, this concern, maybe you should talk to the patient’. Er, then maybe you ought to set up some sort of communication with the doctors, etc., for them to review that information and then come back to me either by phone or via electronic means or call me back to clinic.” (PSC patient 1)*  *“You can speak to them, of course, by phone, it could be maybe a letter, maybe perhaps letters from the doctors that said, you know, liver clinic, this is what we’re going to do, the plan of action, even by email… etc., etc. I’m happy with such. Because sometimes the wait until the next clinic might be six months, might be one year, so by then you’ve forgotten that information or maybe it’s not applicable any more….” (PSC patient 1)*  *“Message me, phone me or sent me a letter, I don’t mind” (Transplant patient)* |
| Managing problems or queries relating to PROMs |  | *“Well the best way to do that is a specialist nurse to take that on board, because they’ll be better than the doctors, we have a specialist nurse now, who is an auto-immune liver disease nurse, so she’s someone who could be helpful. We have research nurses; we have transition nurses … So that’s probably the best way to address those kinds of problems.” (PSC doctor)*  *“I had a problem filling it in, I would ask someone in the, like in the team or in the department to help me like, or I would even ask Dr [name] himself, look a blank, oh I don’t understand this, what does it mean, and, I would just ask” (Transplant patient)* |
| Patient-related factors | Patients with learning disabilities and special needs | *“Those that have learning disabilities, it’s getting their parents to do it with them” (PSC doctor)*  *“And then with someone with special needs, again you have to decide who is going to advocate for them (agreement) because, I mean you want to get a true … opinion” (MDT FG participant)*  *“Yes. So, from that point of view. I would think – I mean, me, I would think, I am okay with computers but someone for example with dyslexia or learning needs or doesn’t have access to computers I would say for them that would be an issue. Like I said, even me – who I think is computer-literate – for me, I was lazy, printing it and scanning back in the computer. But, yeah…” (PSC patient 1)* |
|  | Language difficulties | *“And I guess it may be useful for those patients, for whom English is not their first language, to have some of these questions and there, available in their own language.” (PSC doctor 2)*  *“There are some people that we see who require an interpreter, for example, when they come to clinic.” (PSC doctor 2)* |
|  | Boredom | *“Because once they start getting too much, they just get bored.” (PSC doctor)*  *“I wouldn’t mind but I don’t, I don’t know, it might get a bit boring [laughs], you know what I mean?” (Transplant patient 5)* |
|  | Presence of an observer | *“I suppose, and obviously…..if … there’d be certain questions that I wouldn’t want somebody to fill in if they felt there was an observer.” (MDT FG participant)* |
|  | Hygiene issues with clinic ipads | *“I have to be very aware of hygiene and stuff so if someone has just used that iPad with a cough or cold I would be very reluctant to use that one.” (Transplant patient 3)* |
