# Supplementary material for: Patient and clinician opinions of patient reported outcome measures (PROMs) in the management of patients with rare diseases: a qualitative study
Source: Health Qual Life Outcomes. 2020 Jun 10;18:177. doi: 10.1186/s12955-020-01438-5 (PMC7288678; doi:10.1186/s12955-020-01438-5)
Supplement: Supplementary file 7 — Additional file 7: Table S4. Facilitators and Barriers. [file 12955_2020_1438_MOESM7_ESM.docx]

| **Table S4. Facilitators and Barriers** | | |
| --- | --- | --- |
| **Facilitators** | | |
|  | Clinician enthusiasm | *“So I guess us being enthusiastic about it [laughs].” (PSC doctor 2)* |
|  | Use of computer adaptive testing | *“If you could reduce the size of the questionnaire, depending on their answers, so you know computer adaptive testing, that would be helpful.” (PSC doctor 5)*  *“Sometimes if you’re going through a survey that’s online unless it tells you when you’re going to get to the end you kind of sometimes think am I ever going to get there and then people get fed up.” (PSC doctor)* |
|  | Use of reminders | *“I suppose the main barrier is that people have very good intentions to fill them out and then just forget all you know they’re going to do it and then they don’t fill them in so for most of the point,” (PSC doctor 6)*  *“People say oh I’ll do this survey tomorrow or I’ll do it later and they probably never get around to doing it to be honest…” (Transplant patient)*  *“Obviously, it might take time so better if people do it in advance. People can have a look at it and bring it into the clinic, erm, but yeah, if you send it round and they might forget to fill it in and then it doesn’t become a tool at that point.” (PSC patient 1)* |
| **Barriers** | | |
|  | Lack of awareness | *“They won’t understand how it helps them. You might discover that clinicians don’t think it’s important, because they’ll say, well this is just academic, it’s just a way of measuring something that I know, it’s designed to find out more symptoms than are relevant, so they’ll just say, well I don’t think it’s important.” (PSC doctor 3)*  *“Patients won’t understand why you’re doing it.” (PSC doctor)* |
|  | Access to internet or computer or phones | *“Not everyone will be electronic; they’re not all good at computers, or phones.” (PSC doctor)*  *“Erm…[Laughter] Er, well, er, I prefer a general chat****,*** *to be honest. Erm, rather an electronic questionnaire, but the electronic questionnaire gives you time to think about the, the answer, er, so yes, yes, I think it is a good idea. But not everybody has a computer.” (PSC patient 2)*  *“I know most people have but, but you are talking really about people, er, of a little bit later age that I would think, ‘cos I, I don’t know but I would imagine PSC relates to, to, doesn’t relate to young people.” (PSC patient 2)*  *“I mean, me, I would think, I am okay with computers but someone for example with dyslexia or learning* *needs or doesn’t have access to computers I would say for them that would be an issue. Like I said, even me – who I think is computer-literate – for me, I was lazy, printing it and scanning back in the computer. But, yeah…” (PSC patient 1)*  *“Like it may not load properly or someone might not have a, have access to an electronic device.” (Transplant patient)* |
|  | Non-availability of MyHealth as a mobile phone application | *“Don’t forget MyHealth at the moment doesn’t run on a phone, it’s not an app, so most of our kids are signed up to it but they don’t use it because it’s not an app, so you know they don’t, you know, they just don’t really use it.” (MDT participant)*  *“Cause I don’t think it’s mobile‑friendly yet is it, the, and, you know, even then some people don’t have smartphones” (Transplant patient)* |
|  | Computer literacy | *“Erm, just depends on the audience I guess. Well someone like myself will be fine using electronic and it wouldn’t be a problem because at least you could put it on iPad or you can email it and put it on pdf and they’ll fill it in and send it back or whatever, that’s fine. If you’ve someone who’s 65 or 55 in the clinic and not saying that every 55 but maybe [laughs] some people, maybe some people who are not computer literate maybe [yeah, yeah, yeah, yeah]. They’re not going to really enjoy or really be able to, they wouldn’t be able to do it are they? So that’s, because when I go to clinic I don’t see many young people that if that makes, if that makes sense? So for myself it isn’t going to be a problem but maybe for other people it might be.” (PSC patient 4)*  *“It’s just that I’m not too familiar with computers.” (PSC patient 2)*  *“There will be a group who is not, who aren’t as comfortable, so it’s important to have the other option, as well as the electronic option.” (PSC doctor 5)*  *“Maybe you need, if you’re going to do it electronically you might need to put somebody to support that.” (PSC patient 4)*  *“Maybe, maybe you can do it when, you know, when you do your blood pressure and weight and stuff? Maybe you can get the nurse to just quickly; if it was on an iPad or something she could just quickly go through the questions…” (PSC patient 4)* |
|  | Time constraints | *“But we did something similar in the transplant clinics, we asked them exactly that, about distress, and suicide was on there, and honestly there wasn’t any time to deal with those things.” (MDT participant)*  *“And when you, you know, you need to stop the consultation for, you know, you need half an hour consultation if somebody reveals that they’re feeling suicidal and you know (laughs) in the middle of a big transplant clinic … I just think it needs to be thought out, you can’t just introduce it and then just sort of, it’s like an unexploded bomb then.” (MDT participant)* |
